# Pregnancy health in POWERMOM participants living in rural versus urban zip codes

**DOI:** 10.1017/cts.2020.33

**Published:** 2020-04-06

**Authors:** Jennifer M. Radin, Shaquille Peters, Lauren Ariniello, Shannon Wongvibulsin, Michael Galarnyk, Jill Waalen, Steven R. Steinhubl

**Affiliations:** 1 Scripps Research Translational Institute, La Jolla, CA, USA; 2 Johns Hopkins University School of Medicine, Baltimore, MD, USA

**Keywords:** Rural, urban, maternity, digital health, application

## Abstract

**Background:**

Pregnant women living in rural locations in the USA have higher rates of maternal and infant mortality compared to their urban counterparts. One factor contributing to this disparity may be lack of representation of rural women in traditional clinical research studies of pregnancy. Barriers to participation often include transportation to research facilities, which are typically located in urban centers, childcare, and inability to participate during nonwork hours.

**Methods:**

POWERMOM is a digital research app which allows participants to share both survey and sensor data during their pregnancy. Through non-targeted, national outreach a study population of 3612 participants (591 from rural zip codes and 3021 from urban zip codes) have been enrolled so far in the study, beginning on March 16, 2017, through September 20, 2019.

**Results:**

On average rural participants in our study were younger, had higher pre-pregnancy weights, were less racially diverse, and were more likely to plan a home birth compared to the urban participants. Both groups showed similar engagement in terms of week of pregnancy when they joined, percentage of surveys completed, and completion of the outcome survey after they delivered their baby. However, rural participants shared less HealthKit or sensor data compared to urban participants.

**Discussion:**

Our study demonstrated the feasibility and effectiveness of enrolling pregnant women living in rural zip codes using a digital research study embedded within a popular pregnancy app. Future efforts to conduct remote digital research studies could help fill representation and knowledge gaps related to pregnant women.

## Introduction

Rural pregnant women in the USA and elsewhere typically face many challenges and barriers to access quality prenatal care. For instance, 50% of US counties lack even one obstetrician-gynecologist or hospital with perinatal services [1]. Furthermore, only 49.8% of rural pregnant women lived within a 30-minute drive to the nearest hospital offering perinatal care, compared with 93.4% of urban pregnant women in 2010 [2]. This disparity becomes even more significant for women living in isolated rural areas of which only 28.8% live within a 30-minute drive to the closest perinatal care facility [2]. Additionally, rural pregnant women tend to be underinsured or uninsured and on average are of lower socioeconomic status compared to urban pregnant women [3–6], which further reduces their access to care and may increase their risk of poor health outcomes [6]. These factors contribute to large disparities in infant and maternal mortality rates between rural and urban populations. In the USA, maternal mortality rates in 2015 were 18.2 per 100,000 live births in metropolitan areas versus 29.4 per 100,000 live births in rural areas [7], and in 2014, there were 6.55 infant deaths per 1000 live births in rural populations compared to 5.44 infant deaths per 1000 live births in large urban counties [8].

Pregnant women have historically been left out of research studies, due to scientific, ethical, and legal complexities and fears of harming the fetus [9–11]. For example, a systematic review of pharmacokinetic studies from the late 1960s through 2013 found only 1.29% included pregnant women [12], even though around 70% of women take at least one prescription drug at some point during their pregnancies [13]. This disparity is likely even more apparent for pregnant women living in rural areas due to the distance required to travel to clinical research centers, which are typically located in urban academic centers. With the widespread adoption of digital and mobile technologies, these are new avenues that could be used to fill the void between clinical research and rural pregnant women. Although gaps between rural and urban smartphone ownership exist, 71% of rural populations and 83% of urban populations owned a smartphone in 2019 [14]. This gives most adults the opportunity to participate in digital research platforms remotely from their smartphones regardless of whether they live in urban or rural settings.

POWERMOM is a digital, site-less research study that recruits, enrolls, and obtains data from pregnant women living anywhere in the USA through an iOS app. From March 16, 2017, to September 20, 2019, POWERMOM enrolled 591 participants (16.3% of the total POWERMOM participants) from rural zip codes across the USA. The main objective of this study is to compare the feasibility, practicality, and effectiveness of enrolling and engaging rural pregnant women through a digital study. The secondary objective is to provide a preliminary comparison of pregnancy-related health metrics between urban and rural POWERMOM participants.

## Methods

### Participant Recruitment

POWERMOM was initially developed using Apple’s ResearchKit framework [15] and embedded within WebMD’s iOS Pregnancy App. In addition to the ResearchKit app, a stand-alone app called POWERMOM has recently been deployed, which includes the same study tasks, but makes the app accessible to Android and web users (however, this manuscript only includes data collected on the original iOS platform).

Recruitment messaging was done within the nonstudy portion of WebMD’s pregnancy app which urged users to click on the study section of the app, as well as an advertisement in the WebMD magazine. Participants were not specifically recruited from rural or urban zip codes.

In order to be eligible for POWERMOM, participants had to be 18 years or older, reside in the USA, be comfortable reading and writing on an iPhone in English, and be pregnant at the time of enrollment. Participants that passed the screening questions were then asked to complete the electronic consent process and registration which has been previously described [16]. Ethical oversight of the study was obtained from the Scripps Institutional Review Board.

### Surveys

After the e-consent and registration, participants were asked to complete a series of surveys in the app. All survey questions provided the option to skip the question. The first intake survey asked participants for the date of birth, race and/or ethnicity, zip code, height, weight before pregnancy, current weight and blood pressure, and the date and locations of the measurements. Two days after enrollment in the study, the participant is assigned a health history survey. In this survey, participants were asked additional questions about their pregnancy, prenatal care, and overall medical history. After this initial health history survey, participants were then sent weekly surveys throughout the course of their pregnancy. Weekly surveys asked participants if they were still pregnant or if they had a miscarriage or delivered, their weight, blood pressure, pulse, symptoms such as nausea and vomiting, new medications or vaccines, and additional prenatal visits. If a participant selected that they gave birth in the weekly survey, this triggered an outcome survey to be loaded in their app activities. The outcome survey asked when the baby was born, baby’s weight and length, sex, whether labor was induced and if an epidural was used, type of delivery (vaginal or C-section), and where the birth took place. Users who did not submit the outcome survey 4 weeks after their due date were sent a notification message to remind them to answer the questions to complete their participation in the study.

### Data Collection and Statistical Analysis

**Fig. 1.**
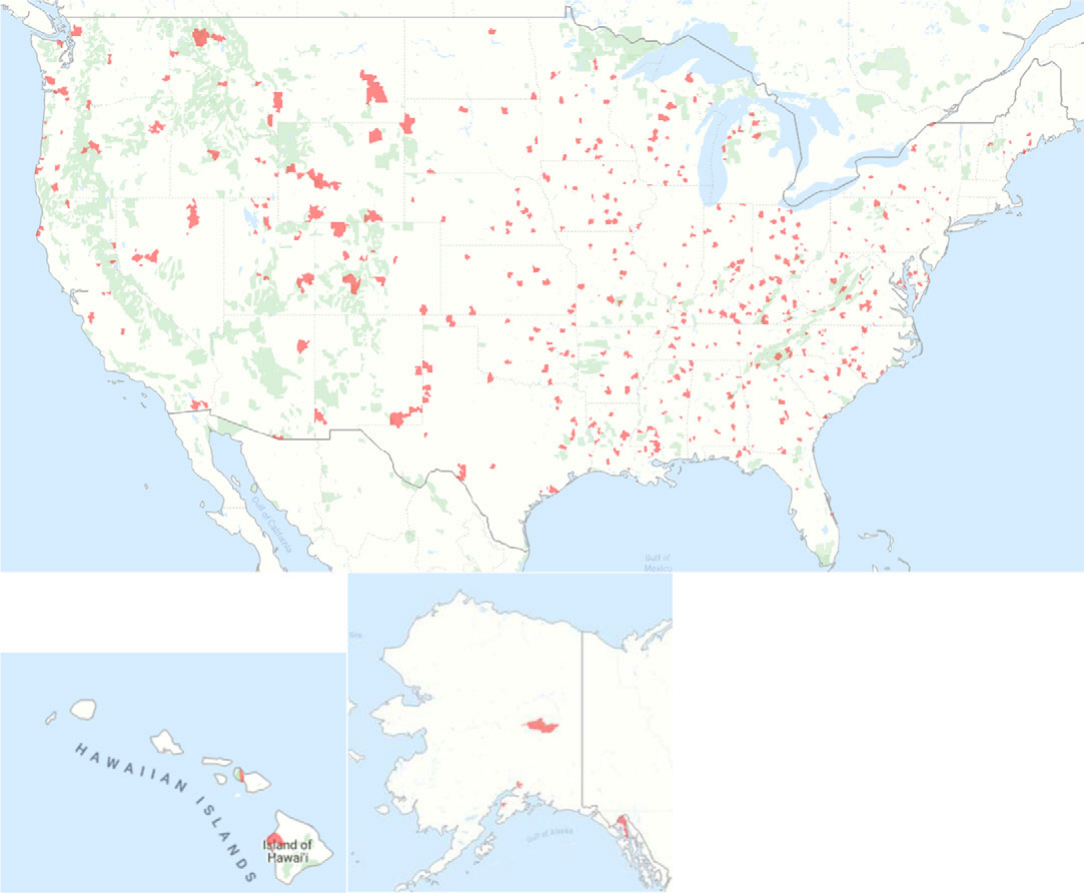
Map of rural participant’s zip codes (in red), March 16, 2017−September 20, 2019 *n* = 591.

Data for this study were collected from March 16, 2017, to September 20, 2019. We excluded participants who were under 18 years old, had missing birth dates, missing due dates, joined the pregnancy study during a pregnancy week that was less than 0 or greater than 41, had a missing zip code, or zip code not represented in the Metropolitan Statistical Area (MSA) coding scheme. Statistical analysis for this study was performed using SAS version 9.4 and R version 3.5.1. The map in Fig. 1 was created using Maptive.

Individuals were classified as urban or rural participants based upon their zip code. The MSA coding scheme was used for the classification of urban versus rural zip codes. Individuals with missing zip code information were excluded from the analysis. Zip codes with an MSA code equal to zero were considered rural and the rest were considered urban. Individuals with zip codes not included in the MSA coding scheme were excluded from this analysis.

Engagement was measured by the number of weekly surveys completed by each participant. Weight changes (current weight – pre-pregnancy weight) and blood pressure (systolic/diastolic) measurements were determined from self-reported weekly surveys and plotted as a function of pregnancy week. Only systolic blood pressure values and diastolic blood pressure values greater than 60 and 30 mmHg, respectively, were considered. Absolute values of weight changes greater than 100 pounds were excluded.

## Results

From March 16, 2017, to September 20, 2019, 4014 participants were enrolled in our study, out of which 69 were excluded for having a missing age, 165 for being less than <18 years old, 85 for having a missing due date, or pregnancy week when joined that was less than 0 or greater than 41 weeks, and 160 for having a missing zip code or zip codes not included in the MSA coding scheme. After these exclusions, there were a total of 3612 participants of which 591 were from rural zip codes (Fig. 1) and 3021 from urban zip codes.

On average participants living in the rural zip codes were younger with a mean age of 28.5 years compared to the urban participants who had a mean age of 30.8 years. The urban population had greater racial diversity compared to the rural population which was 87.8% white. Participants in the rural population also had significantly higher pre-pregnancy weights (165.4 lbs.), compared to the urban population (160.8 lbs.) and higher percentages of participants who were overweight (27.8%) or obese (27.4%) compared to urban participants. There was no significant difference in the percentage of rural versus urban participants who took prenatal vitamins or who had preexisting conditions. There was also no difference in the type of provider that participants saw, except for a significantly higher percentage of women reporting a homebirth/midwife who live in rural zip codes (2.3%) compared to urban ones (0.9%) (Table 1).

**Table 1.** Comparison of rural versus urban populations (singleton pregnancies), March 16, 2017−September 20, 2019, n = 3612

	Rural (*n *= 591)	Urban (*n *= 3021)	*p*-value
Age (years), mean (SD)	28.5 (5.9)	30.8 (5.8)	<0.0001
Age category			<0.0001
18−19 years	44 (7.5)	108 (3.6)	
20−29 years	310 (52.5)	1178 (39.0)	
30−39 years	225 (38.1)	1589 (52.6)	
40+	12 (2.0)	146 (4.8)	
Race/ethnicity*			
White	519 (87.8)	2218 (73.4)	<0.0001
Black or African American	44 (7.5)	434 (14.4)	<0.0001
Hispanic, Latino or Spanish	38 (6.4)	406 (13.4)	<0.0001
Asian	10 (1.7)	124 (4.1)	0.005
American Indian or Alaskan Native	21 (3.6)	89 (3.0)	0.43
Middle Eastern or North African	0 (0.0)	25 (0.8)	0.027
Pre-pregnancy weight (lbs.), mean (SD)	165.4 (45.4)	160.8 (43.5)	0.02
Pre-pregnancy BMI (kg/m^2^)			0.03
Underweight (<18.5)	24 (4.2)	161 (5.4)	
Normal (18.5−24.9)	234 (40.6)	1363 (45.9)	
Overweight (25.0−29.9)	160 (27.8)	707 (23.8)	
Obese (≥30.0)	158 (27.4)	736 (24.8)	
Prior miscarriage	164 (32.1)	880 (33.1)	0.66
Preexisting condition(s)			
Anxiety and/or depression	104 (20.2)	529 (19.8)	0.83
Hypertension	23 (4.5)	105 (3.9)	0.56
Eating disorder	16 (3.1)	85 (3.2)	0.94
None	438 (84.9)	2286 (85.2)	0.85
Prenatal vitamins (Yes)	474 (91.9)	2509 (93.6)	0.15
Healthcare provider			
None	15 (2.9)	85 (3.2)	0.75
Nurse/midwife	79 (15.3)	414 (15.4)	0.94
Home birth/midwife	12 (2.3)	24 (0.9)	0.005
Obstetrician	445 (86.2)	2288 (85.3)	0.57

* Women may identify more than one race/ethnicity.

For both rural and urban groups, the mean week of pregnancy when participants joined the study was approximately 16 weeks. The number of surveys filled out did not vary between rural versus urban groups; on average, participants from both groups completed six weekly surveys, four blood pressure measurements, and five weight measurements during their participation in the study. There was also no significant difference between engagement in completing the outcome survey; 95.2% of rural participants and 89.3% of urban participants completed their outcome survey by 4 weeks past their due date. Overall, higher percentages of participants from urban zip codes compared to rural zip codes shared HealthKit data, with a significantly higher amount for blood pressure and weight (Table 2).

**Table 2.** Engagement of rural versus urban populations, mean (SD), March 16, 2017−September 20, 2019, n = 3612

		Rural (*n* = 591)	Urban (*n* = 3021)	*p*-value
Joined study	Pregnancy week, mean (SD)	15. 8 (10.4)	15.8 (10.7)	0.87
Survey data	No. of weekly surveys, mean (SD)*	6.0 (7.0)	6.0 (7.1)	0.93
	Average no. of BP measurements, mean (SD)**	3.9 (4.5)	4.0 (4.4)	0.60
	Average no. of weight measurements, mean (SD)***	5.2 (6.0)	5.3 (6.3)	0.70
	% of Participants who completed outcome survey****	95.1%	89.2%	0.37
HealthKit data	No. of participants who shared data (% of Total)			
	Steps	244 (41.3)	1348 (44.6)	0.14
	Blood pressure	8 (1.4)	89 (2.9)	0.03
	Sleep	47 (8.0)	287 (9.5)	0.23
	Weight	32 (5.4)	237 (7.8)	0.04

* *n* = 493 rural participants, *n* = 2611 urban participants completed one or more weekly surveys.

** *n* = 347 rural participants, *n* = 1739 urban participants recorded one or more blood pressure measurements.

*** *n* = 469 rural participants, *n* = 2466 urban participants recorded one or more weight measurements.

**** Out of the participants who reached 4 weeks past their due date. (*n* = 42 for rural, *n* = 261 for urban).

**Fig. 2.**
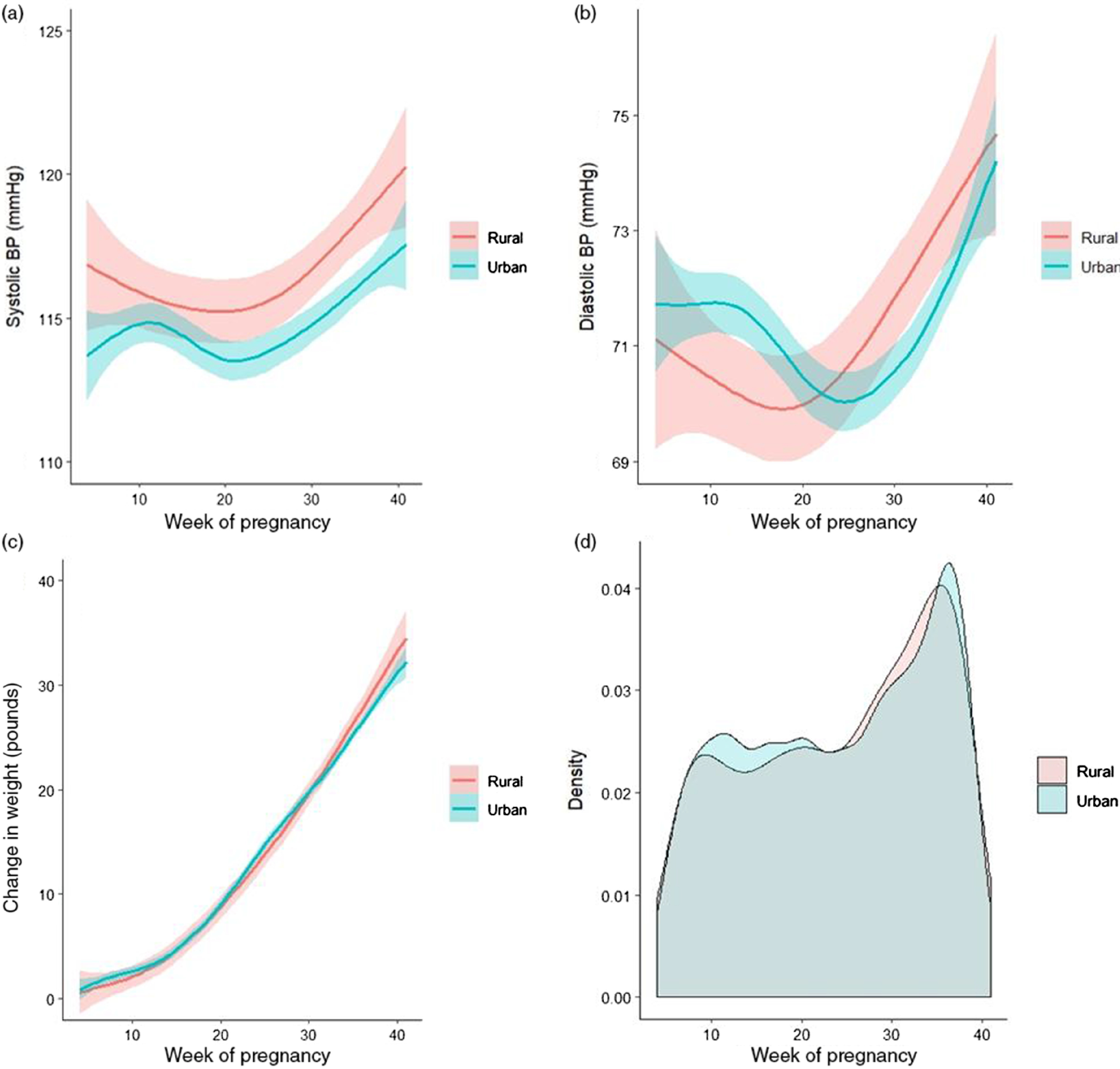
(a) Systolic blood pressure (BP), (b) diastolic blood pressure, and (c) weight change by week of pregnancy (from 4 to 41 weeks) between urban and rural participants. Smooth curves show the smoothed conditional means for each group. The surrounding bands show the 95% confidence intervals around each curve. (d) Density plot comparing pattern of urban versus rural participants recording blood pressure over time (between 4 and 41 weeks of pregnancy).

There was a trend of increasing blood pressure over time as pregnancy progressed for both urban and rural participants. For the systolic blood pressure, the increase was on average greater for the rural participants than for the urban participants, especially toward the latter part of the pregnancy. There was increasing engagement with blood pressure measurements by participants toward the latter part of the pregnancy. For weight change over time, there was also an increasing trend for both groups that does not differ significantly (Fig. 2).

## Discussion

Rural populations have typically been underrepresented in research studies. For example, an analysis from the National Cancer Institute found that only 3% of its research focused on rural populations [17]. Some major barriers that prevent rural populations from participating in clinical trials include lack of knowledge about clinical research opportunities, limited transportation to research centers which are typically located at urban academic research institutes [18], lack of childcare during participation, and difficulty in obtaining time off work to participate [19]. This lack of representation may contribute to worse health outcomes of rural versus urban pregnant women. Our study demonstrates the potential of using an app-based platform to enroll and collect useful pregnancy health data from rural populations across the USA. It is important to recognize that the POWERMOM study did not actively pursue a rural population, suggesting with more targeted outreach, even greater representation is possible. Future incorporation of digital clinical research tools could help increase representation of this population and also serve as a way to engage participants in their own health through visualizations and data feedback.

There were some key differences in participant characteristics among the rural versus urban groups. Rural participants in POWERMOM were significantly younger than our urban population (2.3 years younger on average). Similarly, data from the National Vital Statistics System found that in 2017, the mean age at first birth was 24.5 years in rural counties, 25.8 years in small or medium metro counties and 27.7 years in large metro counties [20]. Our study also had higher percentages of white participants and less overall diversity among the rural zip codes compared to the urban ones. This is comparable to other studies which found women giving birth in rural hospitals were more likely to be white compared to urban populations [21].

Our rural study population also had a significantly larger proportion of overweight and obese participants compared to the urban population. Likewise, other research studies have found that rural pregnant women had higher odds of being overweight or obese compared to non-rural women 22]. Both rural and urban groups reported gaining about 30+ pounds during their pregnancy. American College of Obstetricians and Gynecologists (ACOG) guidelines recommend women who are normal weight gain 25−35 lbs. and women who are overweight or obese gain 15−25 lbs. or 11−20 lbs., respectively [23]. Given that the rural population had higher rates of obesity, there are likely more women in this group gaining above the recommended weight gain. Similarly, a study in rural Pennsylvania found that the majority of overweight and obese women gained above the recommended Institute of Medicine’s Guidelines for weight gain during pregnancy [24].

A study looking at data from the National Inpatient Sample data from 2005 to 2014 did not find any significant differences in chronic hypertension or other chronic conditions among hospitalized deliveries among rural versus urban zip codes [25]. Likewise, we did not see any significant difference in reporting of any preexisting conditions or hypertension between the two groups. However, we did see overall higher self-reported systolic blood pressures among the rural population which was significantly higher than the urban population during some weeks of pregnancy (Fig. 2). The higher blood pressure measurements among the rural population may be a result of higher rates of obesity or perhaps insufficient treatment of hypertensive disorders during pregnancy. We also did not see significant differences in eating disorders or anxiety and/or depression. Likewise, other studies have not found a significant difference in depression between rural and large metropolitan areas but have not specifically looked at differences in pregnancy populations [26].

Interestingly, our rural and urban study population reported high use of prenatal vitamins (>90%) which is much higher than the 2004 Behavioral Risk Factor Surveillance System which found 78% of pregnant women took multivitamins [27]. This may be a result of selection bias, with users of the WebMD pregnancy app potentially being more engaged and informed about their health and more likely to use a research app. Additionally, since our app was initially only available to iOS users, it might have selected users who were wealthier and more likely to afford prenatal vitamins compared to the general US population.

Both rural and urban groups reported seeing similar types of healthcare providers for their pregnancy and delivery, with the majority of women in both groups seeing an obstetrician. However, there was a significantly higher percentage of women planning to have a homebirth/midwife at their delivery who lived in rural zip codes (2.3%) compared to urban zip codes (0.9%). The percentage of home births in rural zip codes is higher than the average of 1.6% in the USA in 2017 [28]. This larger percentage of home birth may be a reflection of increasing drive times to hospitals with perinatal care in the USA [2].

We found that pregnant women living in rural zip codes had similar engagement within our study. Interestingly, both groups joined the study around the same time in their pregnancy and stayed engaged for a similar amount of time. A higher, but not significantly different percentage of participants filled out the outcome survey in the rural population compared to the urban population. There was a higher percentage of urban participants who shared HealthKit data, which may reflect access to digital devices such as Apple watches to measure activity, and heart rate and home blood pressure cuffs and weight scales. Since participants had to utilize their own digital devices, future efforts to supply these sensors to underrepresented populations will be important.

One major strength of our research platform is that it is embedded within WebMD’s pregnancy app, a widely used resource for pregnancy-related information, which helped provide trust and visibility in enrolling a relatively large and diverse population. Since our study was entirely digital, it removed some of the barriers associated with participation in traditional research such as finding suitable times to participate and transportation to research sites. However, a major limitation of the first version of the app (summarized in this study) was that it was only available in iOS (although Android and HTML have recently been added). iOS smartphone users are typically more affluent than Android users [29] and may be more prevalent in urban regions rather than rural ones. This may have reduced the opportunity for rural participants to engage in our study. Additionally, it is possible that our existing data underpowered to identify some differences between rural and urban groups. Despite this, we were still able to enroll and engage a large percentage of rural participants from across the country.

In conclusion, we found that there were some key differences between rural and urban pregnancy populations, such as lower age and higher rates of obesity. However, both groups had similar engagement and retention using our digital platform. This may indicate a willingness by rural populations to participate in clinical research studies given the opportunity to share their data remotely during times that are self-selected and convenient. Increasing representation of underrepresented populations in clinical research is important for improving our understanding of disease burden among rural populations and designing future interventions to improve health outcomes.
